# Detection of Executive Performance Profiles Using the ENFEN Battery in Children Diagnosed With Attention-Deficit Hyperactivity Disorder

**DOI:** 10.3389/fpsyg.2020.552322

**Published:** 2020-12-07

**Authors:** Ignasi Navarro-Soria, Rocío Juárez-Ruiz de Mier, José Manuel García-Fernández, Carlota González-Gómez, Marta Real-Fernández, Marta Sánchez-Múñoz de León, Rocío Lavigne-Cervan

**Affiliations:** ^1^Department of Developmental Psychology and Didactics, University of Alicante, Alicante, Spain; ^2^Department of Developmental Psychology and Education, University of Málaga, Málaga, Spain; ^3^Department of Health Psychology, Miguel Hernandez University of Elche, Elche, Spain

**Keywords:** ENFEN, WISC-IV, executive function, ADHD, ADHD rating scale

## Abstract

Attention-deficit hyperactivity disorder (ADHD) is one of the most common neurodevelopmental disorders in children and adolescents. People who have this disorder are characterized by presenting difficulties in the processes of sustained attention, being very active, and having poor control of their impulses. Despite the high prevalence of this disorder and the existence of various tests used for its diagnosis, few data are available regarding the usefulness and diagnostic validity of these tools. Given the difficulties that these subjects present in executive functions, the aim of this study was to evaluate whether the Neuropsychological Assessment of Executive Functions battery for Children (ENFEN, for its acronym in Spanish, Portellano et al., 2009) allows to establish specific profiles of executive performance for people with attention-deficit hyperactivity disorder (ADHD). The sample was made up of 197 participants of both sexes, aged between 6 and 12 years age (134 with a clinical diagnosis and 63 without pathology). A nonexperimental design was followed, using a comparative descriptive study method. The results indicated that the scales of phonological fluency, color path, rings, and interference are the most associated with the diagnosis of ADHD, providing data on inhibition, mental flexibility, sustained and selective attention, planning, verbal fluency, and working memory, among others. The practical implication of these results is in line with providing support in the clinical diagnosis that is carried out in children’s mental health units. In addition, the ENFEN tool can be valued as a suitable psychometric instrument in the psychoeducational field, helping professionals in a school environment to be more aware of the areas of cognitive development in which a student diagnosed with ADHD will have more difficulties and, in doing so, providing more adjusted and effective psychopedagogical measures when it comes to supporting students in their adaptation to the school environment.

## Introduction

The term executive functions (EFs, hereinafter) is a relatively recent construct, developing its object of study in areas of knowledge such as neuroscience, neuropsychology, or psychiatry. In recent decades, these disciplines have undergone considerable evolution due to the development of successive theoretical models from cognitive psychology, as well as the advances made in regard to studying brain activity (Feinberg and Farah, 1997). This fact has favored the growing interest in the understanding of these mental capacities, as well as in the study of their neuroanatomical bases (Tirapu et al., 2002). Over the past decades of research, EFs have been linked to the frontal lobes, and their dysfunction, to frontal lobe syndrome (Bausela-Herreras et al., 2019). The prefrontal cortex constitutes the part of the human being that differentiates us most from other species, being the brain region with more recent phylogenetic and ontogenetic development and playing a fundamental role in complex and evolved cognitive functions (creativity, social behavior, decision making, moral judgment, etc.; Goldman, 1984; Price et al., 1990; Pelegrín and Tirapu, 1995; Fuster, 1997).

In 1980, Luria proposed three functional units in the human brain: (a) alertness/motivation, (b) reception/processing/storage of information, and (c) programming/control/verification of brain activity (this third function would depend directly on areas of the prefrontal cortex and would play an executive role; (Ardilla and Ostrosky, 2008). However, it was Lezak who would use the term for the first time in 1982, when referring to the mental capacities necessary to carry out an effective, creative, and socially accepted behavior, needing four main components: formulation of goals, planning of actions and sequences to achieve a goal, development of planned actions and execution, and monitoring and correction of behaviors (Luria, 1980; Lezak, 1982).

Tirapu et al. (2002), with the aim of elaborating and integrating proposal that gathered the foundations of EFs, carried out an excellent work combining elements from Baddeley’s working memory (WM, hereinafter) models, the hierarchical functions of Stuss and Benson, the supervisory attentional system (SAS, hereinafter) from Shallice, and the somatic marker hypothesis of Damasio. WM is related to sensory and perceptual components; and in the face of novel actions in which there is no known solution and decisions need to be made, it requires the participation of the SAS to kick-start anticipation, planning, and control processes. Therefore, the cognitive and behavioral responses in this type of situation are not automatic or overlearned but configured based on the analysis that the person carries out regarding the consequences of their own actions. In all of this, the somatic marker forces the attention and WM of the individual towards the possible effects, both positive and negative, of such non-automatic actions. This model proposes that EFs constitute an “extended system,” where the SAS and WM operate with the available information to generate alternatives, while the somatic marker forces the cognitive resources (attention and WM) towards one alternative (the most adaptive), based on previous representations of positive and negative states, generating successive approximations (in neural terms) through processes of anticipation, selection of objectives, planning, and control.

At present, EFs are usually defined as high-level cognitive functions that are oriented to problem solving, goal planning, and the achievement of complex objectives, in the face of novel and nonroutine situations that involve adaptive responses (Lavigne, 2009; Lavigne and Romero, 2010a). However, there is no consensus when delimiting their nuclear dimensions, which complicates its operationalization (Lavigne, 2009; Bausela-Herreras et al., 2019).

There are numerous neurological pathologies and mental disorders in which damage to components of executive functioning has been described (Tirapu et al., 2002). Specifically, Artigas (2011) refers to neurodevelopmental disorders (American Psychiatric Association, 2013) as delays in the development of EFs linked to the maturation of the central nervous system, which starts in childhood and follows a stable evolutionary course. These disorders do not present biological markers for their detection, with comorbidity being the most frequent form of presentation and limits between them possibly being imperceptible (Gupta and Kar, 2010; Bausela-Herreras et al., 2019). There are various neurodevelopmental disorders that, despite being independent diagnostic entities, share common manifestations regarding alterations in EF: attention-deficit hyperactivity disorder (ADHD), autism spectrum disorder (ASD), specific learning disorder, language disorders, or behavioral disorders, among others (Henry et al., 2015; Craig et al., 2016; Huang et al., 2016; Shuai et al., 2017).

Lavigne and Romero (2010b) elaborate an operative and explanatory definition of executive functioning from an ADHD brain dysfunction point-of-view. They start by defining the executive system (ES) as an information processing system integrated by anatomical and psychological components on which the EFs depend. The structural components refer to central nervous system areas, mainly frontal lobes, as well as to the cerebellum and other subcortical structures (basal ganglia, limbic system, and amygdala) and to the neural circuits that innervate the connections between them (Barkley, 1997, 2006a,b, 2009; Fuster, 1997; Frazer et al., 1999; Pineda, 2000; Bradley and Golden, 2001; Brown, 2003; Goldberg, 2004; Martín et al., 2008). WM and its relationship with the anterior attention system, language and its internalization, socio-emotional self-regulation (Sánchez et al., 2019), and the processes of analysis and synthesis constitute the central psychological processes in the ES of people with ADHD. The EFs (planning, organization, self-monitoring, cognitive flexibility, persistence, and evaluation) depend directly on such structures and processes and exercise a conscious, voluntary, and final control over human behavior. There is a certain consensus in researchers who study the EFs and their relationship with ADHD regarding the central role they play in cognitive and behavioral control, as well as in the anatomical bases and psychological processes on which they depend (Gupta et al., 2006; Gupta and Kar, 2009; Lavigne, 2009; Lavigne and Romero, 2010b; Gupta et al., 2011; Tirapu et al., 2017). The effort of various authors to facilitate the understanding and functioning of the ES is oriented, among other purposes, towards the development of precise evaluation protocols and effective stimulation programs that enhance the affected variables.

However, the evaluation of deficits in EFs is an especially problematic process due to the fact that many batteries and neuropsychological tests [Wisconsin Card Sorting Test (WCST), Stroop, Trail Making Test, phonetic verbal fluency, etc.] show sensitivity in identifying frontal dysfunctions and are unspecific in measuring alterations in the ES. The validity of these batteries in the evaluation and diagnosis process is diminished by the lack of operability in the description of EFs and the structure of the batteries themselves, as well as the artificiality of the tests that compose them (Tirapu et al., 2002).

The broad spectrum of ADHD and its high comorbidity, together with the difficulty in conceptualizing EFs, has created the need to establish more precise diagnostic protocols. Abad-Mas et al. (2017) point out the importance of carrying out a complete neuropsychological evaluation of the superior brain functions for an adequate definition and detection of the disorder, considering the symptoms described in the DSM to be imprecise for a formal diagnosis. Along these lines, studies that define cognitive patterns in neurological disorders seem to facilitate detection and discrimination between pathologies, aiding in a reliable diagnosis and a choice of an appropriate therapeutic treatment (Thaler et al., 2010; Kasper et al., 2012; Mattison and Mayes, 2012; Qian et al., 2013; Fenollar et al., 2015).

With the objective of finding cognitive profiles that help diagnose ADHD, Navarro et al. (2020), using the Wechsler Intelligence Scale for Children IV Edition (WISC-IV), found significant differences as compared with a control group in the Cognitive Proficiency Index (CPI, hereinafter), while the General Ability Index (GAI, hereinafter) turned out to be similar between both groups. Therefore, the scores obtained on the WM and processing speed (PS) indices, which make up the CPI, allowed for discriminating a cognitive phenotype of people with the disorder (Devena and Watkins, 2012; Thaler et al., 2013; Fenollar et al., 2015). Navarro et al. (2020) concluded that a cognitive step could be found between GAI and CPI, not when low scores were obtained in WM or PS, but when the subject achieved lower scores in these areas in comparison with other indices.

On the other hand, Bausela-Herreras et al. (2019) observe that in most of the studies carried out on people with ADHD, deficits are found in aspects of executive functioning such as inhibition, WM, cognitive flexibility, and planning. However, in most of said studies, independent scales, long-term tests, or tests that do not have their corresponding Spanish adaptation are used. Furthermore, in the current literature, there is great difficulty in finding instruments that can be applied to children, with the majority being administered in adolescents or adult population (Peña-Casanova et al., 2012; Rognoni et al., 2013).

Therefore, EFs are mediated by the prefrontal cortex and demand a higher and more complex cognitive-behavioral requirement than is commonly needed to perform goal-orientated behavior. One of the main problems that ADHD subjects present is the difficulty in inhibitory control and the organization of tasks that require a set of consecutive steps (Yasumura et al., 2019), which leads to them showing deficiencies in EFs, the cognitive aptitude that the ENFEN battery requires, in a greater or lesser extent, in each subtest (Portellano et al., 2009). This instrument allows to evaluate EFs and has been widely used in clinical and school contexts, allowing the detection of possible maturational delays or cognitive alterations derived from brain damage or dysfunction, especially in prefrontal areas (Portellano, 2014; Navarro et al., 2019). The ENFEN has been used on numerous occasions to assess the effects of an intervention program (both in ADHD and other disorders or population without pathology; Fernández et al., 2013; Gallego et al., 2015); however, whether a certain disorder can lead to the appearance of a specific response profile has not been studied.

The diagnosis of ADHD is a complex process, with no known biological markers and with the current psychometric tools only providing rough information about the presence of deficiencies in cognitive skills associated with ADHD. Therefore, finding an effective tool to measure these symptoms or cognitive skills is one of the most solid strategies in order to support the diagnosis of the disorder. Traditionally, health professionals have used instruments to measure frequency of appearance of symptoms; however, it is becoming increasingly common to find the use of tests that measure cognitive abilities related to ADHD deficiencies.

Studies that have tried to establish possible cognitive patterns to help the diagnosis of ADHD are scarce, most of them being carried out with batteries that evaluate general cognitive aptitudes. For this reason, the aim of the present study was to verify if the ENFEN battery allows to establish specific executive performance profiles for subjects with ADHD. The specific objective consisted of studying the existence of differences between the executive performance profiles measured with the ENFEN battery, between a group of subjects with a normalized development and two other groups of subjects with ADHD, one with a manifest “cognitive step” and another in which the “cognitive step” is nonexistent, understanding the concept “cognitive step” as the unusual fluctuations, in comparison with oneself, in scores on cognitive tests that require competence in skills usually affected by ADHD such as EFs, WM, and speed of processing information (Fenollar et al., 2015; Navarro et al., 2020).

## Materials and Methods

### Participants

The sample was made up of 197 children, aged between 6 and 12 years (8.53 ± 1.92), from six public schools (four from Alicante and two from Málaga) and two charter schools (Málaga). One group (*n* = 63, 8.78 ± 1.93 years, 37 boys and 26 girls) presented no pathology. Another group (*n* = 134, 8.41 ± 1.92 years, 82 boys and 52 girls) was diagnosed with ADHD, of which 72 (8.50 ± 1.83 years, 43 boys and 29 girls) had a “cognitive step” in the WISC-IV test and the rest did not (*n* = 62, 8.31 ± 2.02 years, 39 boys and 23 girls).

The inclusion criteria for the selection of the groups were as follows:

Normotypic development group (NDG): (a) failure to meet the ADHD diagnostic criteria according to the ADHD Rating Scale DSM-IV and (b) presenting a general capacity index, measured with IV version of Wechsler Intelligence Scale, greater than 80 (Navarro et al., 2017, 2019).

ADHD group with a cognitive step: (a) presenting an ADHD diagnosis taking into account the criteria of the ADHD Rating Scale DSM-IV, (b) presenting a general capacity index, measured with the IV version of Wechsler Intelligence Scale, greater than 80 (Navarro et al., 2017, 2019), and (c) presence of a “cognitive step” determined by a significant difference between the scores for GAI and CPI. Following previously stated research, the presence of this “cognitive step” indicates a higher degree of ADHD, as well as more acute symptoms. For this reason, we differentiate between subjects in the clinical group who present higher or lower severity of symptoms.

ADHD group without a cognitive step: (a) presenting an ADHD diagnosis taking into account the criteria of the ADHD Rating Scale DSM-IV, (b) presenting a general capacity index, measured with IV version of the Wechsler Intelligence Scale greater than 80 (Navarro et al., 2017, 2019), and (c) absence of a “cognitive step” determined by the difference between scores on the GAI and the CPI.

### Instruments

#### ADHD Rating Scale DSM-IV (RS) Scale (DuPaul et al., 1998)

This instrument evaluates, in family and school settings, the dimensions of inattention and impulsiveness/hyperactivity by analyzing the frequency of appearance of nine symptoms in each domain. The Spanish adaptation of this scale was used (Servera and Cardó, 2007), taking a ≥P90 score as clinically significant. The scale consists of 18 Likert-type items (to be completed by parents and teachers), with a scale from 0 to 4 points, where 0 and 1 mean that the symptom never or rarely manifests and 2 and 3 mean that they occur often and/or very often. It is based on the DSM-IV ADHD diagnostic criteria. In the inattention dimension, Cronbach’s alpha coefficients were 0.85 for family members and 0.91 for teachers and in the hyperactivity/impulsivity dimension 0.90 for family members and 0.93 for teachers.

#### Wechsler’s Intelligence Scale for Children, 4th Edition (Wechsler, 2011)

This tool explores general intellectual ability. It is a cognitive test for children between 6 and 16 years of age. The Spanish version (Corral et al., 2005) was used to assess full scale IQ, using 10 subtests (Similarities, Vocabulary, Comprehension, Cubes, Concepts, Matrix Reasoning, Digit Span, Letter-Number Sequencing, Coding, and Symbol Search) grouped into four general indices [Verbal Comprehension Index (VCI), Perceptual Reasoning Index, WM Index (WMI), and PS Index]. In addition, it has five complementary subtests (Information, Comprehension, Picture Concepts, Arithmetic, and Cancellation) that can replace any of the mandatory tests if they cannot be applied for any reason or enrich the exploration by providing additional information. It also offers the GAI and CPI. The GAI is a composite score, based on some of the verbal and nonverbal subtests used to calculate the VCI and the Visual Spatial Index (VSI). On the other hand, the CPI summarizes the results of the WMI and the Processing Speed Index (PSI) in a single score. It is applied individually, and its duration is approximately 90 min.

#### Neuropsychological Assessment of Executive Functions Battery for Children (ENFEN; Portellano et al., 2009)

This battery of tests allows a global evaluation of EFs in children between the ages of 6 and 12. Specifically, it analyzes aspects such as vocabulary range, cognitive flexibility, graphomotor and visuomotor coordination, WM, planning and sequencing capacity, inhibition capacity, or resistance to interference. It is applied individually, and its duration is approximately 20 min. It is made up of four tests (fluency, paths, rings, and interference) divided into six scales. Lastly, a total score for each task was obtained, and raw scores were transformed into sten scores (*M* = 5.5, *SD* = 2).

Fluency task assesses participant’s ability to produce language under time pressure, although it can be an indirect measurement of verbal memory and WM. The child has 1 min to produce as many words as possible from a category: in phonemic fluency, the category was related to words starting by the letter “M” and in semantic fluency “animals.”

Trail Making Test assesses different aspects of EF: flexibility, thinking strategies, inhibition, WM, and executive attention. In the graytrail, the child had to draw a line linking numbers from 20 to 1 that appeared randomly on a sheet of paper. In the color version, the child has to link numbers from 1 to 21, but he/she has to switch among yellow and pink colors.

Inhibition task is derived from the Stroop test, and it is a relatively pure measure of cognitive inhibition, although attention, flexibility, and resistance to interference are also engaged in this task. The child was presented with a sheet that showed three columns with 13 words each. The words were the name of colors (red, green, yellow, and blue) printed with random color inks (red, green, yellow, and blue), with the color name and color ink never matching. The child had to say the color ink of each word.

### Procedure

An associative, comparative, and predictive study method was designed (Ato et al., 2013). For this, a single evaluation was carried out to subsequently determine the differences between the groups analyzed. The sample was selected at various public charter schools and specialized clinics in the municipalities of Málaga and Alicante. First, permission was requested from the management of the respective centers, and subsequently, informed consent was obtained from the parents/legal guardians of the participants who were informed that the data collected would be anonymous and used exclusively for research purposes. The clinical group, diagnosed with ADHD, was gathered from the total sample detected and diagnosed at the collaborating centers. The control group, i.e., student who did not have any difficulties regarding their neurodevelopment, was gathered using convenience sampling. Furthermore, participants could withdraw from participating in the study at any given time. In addition, the ethical principles of the Declaration of Helsinki were respected (World Medical Association, 2013). Finally, approval was obtained from the Ethics Committee of the University of Alicante, assigning it file number UA-2018-03-08.

The evaluations were carried out in the order of the following sequence. First, the ADHD Rating Scale DSM-IV (RS) was completed by parents/legal guardians and teachers, in a single day, with an approximate duration of 10 min each. This battery was used to determine the presence or absence of ADHD symptoms in both the clinical and control samples. A positive diagnosis was corroborated when subjects scored positively on six or more items out of the nine established for each context (family/school). On the other hand, the children were evaluated with the WISC-IV and the ENFEN on two different days, with a duration of 45–90 min approximately. The protocols defined in each instrument were used, with the evaluation taking place individually and in a place free of noise and endowed with privacy.

The groups were formed based on the scores on the ADHD Rating Scale DSM-IV (RS) and the WISC-IV.

### Data Analysis

The data were subjected to descriptive and inferential analyses. The normality was explored by evaluating the skewness and kurtosis of the data set, as well as by the Kolmogorov-Smirnov test. After this, to analyze the differences in the scores between the groups, Kruskal-Wallis analyses were carried out to assess the existence of differences between all groups and the Mann-Whitney U test to specify the differences in pairs. In addition, Cohen’s d (1988) was used to measure the effect size, taking into account the levels determined by Hojat and Xu (2004; ≈0.20, small; ≈0.50, medium, and ≈0.80, large). The SPSS program in its 26.0 version was used for statistical data processing. A binary logistic regression model *(forward* stepwise) was generated to determine the predictive capacity of the ENFEN on the diagnosis of ADHD. As model fit measurements, the Omnibus and Hosmer-Lemeshow parameters were calculated.

## Results

Table 1 shows the descriptive and normality statistics (Kolmogorov-Smirnov) for the ENFEN test scores for each group. As can be observed, the results showed that in most of the cases, data sets were not distributed following the normality criterion. Therefore, the differences between the groups were determined using non-parametric contrast tests.

**Table 1:** Descriptive statistics and Kolmogorov-Smirnov test for the different ENFEN tests and by groups.

		Mean	Standard deviation	Skewness	Kurtosis	Kolmogorov-Smirnov
ADHD (A) (*n* = 62)	Phonological fluency	4.26	1.80	0.45	1.05	0.14^**^
	Semantic fluency	5.58	2.09	−0.08	−0.24	0.13^**^
	Gray path	5.13	2.79	0.18	−0.92	0.11
	Color path	3.34	2.13	0.64	−0.47	0.16^***^
	Rings	4.19	1.29	0.29	−0.47	0.17^***^
	Interference	4.06	2.00	0.24	−0.52	0.12^*^
ADHD (B) (*n* = 72)	Phonological fluency	4.36	2.21	0.32	−0.22	0.09
	Semantic fluency	6.40	2.07	−0.24	−0.90	0.14^**^
	Gray path	4.86	2.48	0.04	−0.82	0.13^**^
	Color path	2.92	1.84	0.64	−0.56	0.18^***^
	Rings	4.10	1.29	−0.31	−0.23	0.17^***^
	Interference	3.71	1.67	−0.01	−0.78	0.15^***^
Normotypic development (*n* = 63)	Phonological fluency	5.78	1.60	0.21	−0.80	0.15^***^
	Semantic fluency	5.92	1.86	0.03	−0.42	0.13^**^
	Gray path	5.68	1.42	0.14	−1.17	0.17^***^
	Color path	5.41	1.68	−0.05	−1.00	0.14^**^
	Rings	5.75	1.78	−0.03	−0.90	0.17^***^
	Interference	5.90	1.77	0.24	−0.55	0.14^**^

(A) Without a cognitive step. (B) With a cognitive step. ADHD, attention-deficit hyperactivity disorder; ND, normotypic development.

* *p* < 0.05.

** *p* < 0.01.

*** *p* < 0.001.

Table 2 shows the comparisons between the three groups (Kruskal-Wallis) and by pairs (Mann-Whitney U). When the differences between groups were significant, the Cohen d value and the confidence interval (95%) were also evaluated. There were no differences between the groups with ADHD, although there were differences between the group without ADHD and the two groups with ADHD in the phonological fluency, path, rings, and interference tests. Results indicated large effect size (Evans, 1996) in those cases where there were statistically significant differences.

**Table 2:** Differences between groups in the analyzed ENFEN tests.

	Kruskal-Wallis	ADHD (A) vs. ADHD (B)		ADHD (A) vs. NDG		ADHD (B) vs. NDG	
		Z	Cohen’s d [95% CI]	Z	Cohen’s d [95% CI]	Z	Cohen’s d [95% CI]
Phonological fluency	24.12^***^	−0.25	–	−4.61^***^	−0.89 [−1.26, −0.53]	−3.95^***^	−0.73 [−1.08, −0.38]
Semantic fluency	5.26	−2.23	–	−0.80	–	−1.46	–
Gray path	4.25	−0.41	–	−1.48	–	−2.01	–
Color path	50.03^***^	−1.05	–	−5.36^***^	−1.08 [−1.46, −0.70]	−6.71^***^	−1.41 [−1.79, −1.03]
Rings	33.93^***^	−0.10	–	−4.86^***^	−1.01 [−1.37, −0.63]	−5.22^***^	−1.07 [−1.43, −0.71]
Interference	41.72^***^	−0.90	–	−4.83^***^	−0.97 [−1.35, −0.60]	−6.20^***^	−1.28 [−1.65, −0.90]

(A) Without a cognitive step. (B) With a cognitive step. NDG, normotypic development group.

*** *p* < 0.001.

A binary logistic regression analysis was performed to determine the diagnosis of ADHD using the ENFEN battery (Table 3). The generated model includes all tests from the ENFEN battery as significant predictors and explains 55% of the variance as a whole (*R*^2^ Nagelkerke = 0.55). This model shows a good fit, correctly classifying 82.7% of cases, with appropriate values from the omnibus test (*χ*^2^ = 99.60; *p* < 0.001) and the Hosmer-Lemeshow test (*χ*^2^ = 13.41; *p* > 0.05). Beta values show a negative value in the equation in the cases of phonological fluency, color path, rings, and interference, as well as positive values in semantic fluency and gray path.

**Table 3:** Logistic regression analysis of ADHD diagnostic criteria regressed on ENFEN factors predictors (forward stepwise).

Criterion	Predictors	B	T.E	Wald	Sig.	Exp (B)
ADHD	Phonological fluency	−0.33	0.15	5.06	0.024	0.72
	Semantic fluency	0.56	0.14	15.17	0.000	1.75
	Gray path	0.45	0.14	11.04	0.001	1.57
	Color path	−0.58	0.15	14.67	0.000	0.56
	Rings	−0.48	0.23	4.42	0.036	0.62
	Interference	−0.41	0.14	8.15	0.004	0.66
	Constant	3.51	0.81	18.51	0.000	33.37

ADHD, attention-deficit hyperactivity disorder.

Regarding the sensitivity and specificity of the different ENFEN tests in their ability to diagnose ADHD, the semantic fluency and gray path tests provide a poor diagnosis with an area under the curve (AUC) of 0.52 and 0.59, respectively, while the phonological test provided a mediocre diagnosis (AUC = 0.71, sensitivity = 73.9%, and specificity 58.7%). The remaining tests gave a good diagnosis but presented large differences between sensitivity and specificity, specifically the color (AUC = 0.81, sensitivity = 63.4%, and specificity 82.5%), interference (AUC = 0.78, sensitivity = 63.4%, and specificity 74.6%), and ring tests (AUC = 0.76, sensitivity = 97%, and specificity 39.5%).

## Discussion

The purpose of this study was to verify if the ENFEN test allows to establish specific profiles of executive performance for subjects with ADHD and thus to demonstrate if there are differences in these profiles between anormotypic group and two other groups of subjects with ADHD, one with a manifest “cognitive step” and another in which the “cognitive step” was nonexistent.

Following Lozano et al. (2015) and Reyes et al. (2014), after analyzing the results of this study, we can affirm that there are significant differences between the group with normotypic development and the groups with ADHD in the execution of the tasks that make up the ENFEN battery, specifically in phonological fluency, color path, rings, and interference. These data support the hypothesis that the ENFEN battery possesses a good ability to discriminate profiles between subjects who have symptoms associated with ADHD and those who do not, whether the presentation of these symptoms are severe or moderate, or the person shows a more or less pronounced “cognitive step” (Fenollar et al., 2015; Navarro et al., 2019). Specifically, the cognitive pattern obtained in this study using the ENFEN battery shows low scores in the phonological fluency, color path, ring, and interference subtests, a significantly lower response pattern compared with the control group and in comparison with scores on the semantic fluency and gray path subtests. In addition, of the three tests that provide an overall good diagnosis of ADHD, the color path test presents a greater AUC and specificity, while rings reflect greater sensitivity. The explanation is that these last two subtests present a lower demand on executive level, which favors subjects with ADHD.

In the case of semantic fluency, classic studies note the existence of different neural systems that are activated in verbal fluency tasks in the face of phonological or semantic instructions, associating the frontal lobe with the first type (Coslett et al., 1991) and the temporary lobe with the second type (Mummery et al., 1996; Baldo et al., 2006). However, there are authors who point out that, although it is true that semantic fluency tasks are sensitive to temporal lobe disorders, both types of tasks – phonological and semantic – show sensitivity regarding the detection of frontal dysfunctions, as they are involved in both executive processes such as initiative, organization, inhibition, and self-monitoring (Henry and Crawford, 2004). As for the gray path task, it presents a remarkable simplicity in comparison with its color version due to the lack of introduction of an interference variable. The requirements of this activity for children from 6 to 12 years old are minimal, with them usually completing the task with great rapidness, therefore consuming less cognitive resources than in color path, rings, or interference. In a similar test to that of color path, Juneja et al. (2019) found that compared with control groups with a normotypic development, people with ADHD also obtained significantly lower performance in its execution.

Other studies have also found relationships between EFs and ADHD using different psychometric measurement scales. The interference subtest that the ENFEN battery presents is derived from its predecessor, the Stroop test (Golden, 2001). Using this test, López-Villalobos et al. (2010) showed that compared with a control group, the group with ADHD showed significantly lower scores, demonstrating their difficulties in flexibility and inhibitory control. In the same way, Pedreira and Peña (2011), using the Five Digits Test (Sedó, 2007), obtained similar results. Ramos and Pérez (2017) conclude from their investigations that subjects diagnosed with ADHD have a higher frequency of errors in the execution of Go/No-Go tasks. Frazier et al. (2004) analyze other tasks that require the use of EFs such as the Rey Complex Figure Test (Rey, 2009), the WCST (Heaton, 1981), or the Trail Making Test (Reitan and Wolfson, 1992), concluding that a poorer performance is observed in the case of subjects diagnosed with ADHD compared with average controls (Gupta et al., 2009, 2011).

In line with the efficacy of the measurement of EFs for the diagnosis of ADHD, other investigations (Mayes and Calhoun, 2006; Thaler et al., 2013; Fenollar et al., 2015; Navarro et al., 2017, 2019), which use a very solid psychometric battery such as the WISC-IV (Wechsler, 2011), conclude that in the case of the clinical population diagnosed with ADHD, their cognitive pattern presents a significant step in the Cognitive Proficiency Index (compared with the GAI, it is an index made up of the scores obtained in Verbal Comprehension and Perceptual Reasoning), an index derived from the global scores obtained in WM and PS, subtests that are more demanding on an executive level (Bustillo and Servera, 2015).

Finally, from the data obtained from the binary logistic regression, it is determined that lower values in variables with a negative sign (phonological fluency, color path, rings, and interference) and higher values in those with a positive sign (semantic fluency and gray path) can be considered as a characteristic sign through which to configure a specific response profile in relation to EFs in the assessment of ADHD. We understand that this is due to the fact that the subtests, in which subjects diagnosed with ADHD obtain higher scores, are less demanding in EFs, which allows them to achieve scores closer to those expected by subjects of the same age who are not affected by this disorder.

Therefore, taking into account all these findings, we can conclude that people diagnosed with ADHD present a characteristic performance profile when executing tasks that demand the proper functioning of the ES and its EFs, as is the case of the ENFEN battery. This unique cognitive profile common to the clinical group in relation to the control group is something that has been under study for more than a decade and presents empirical evidence with various psychometric tools (García et al., 2014).

The practical implication of these results is in line with providing support in the clinical diagnosis that is carried out in children’s mental health units. In addition, the ENFEN tool can be valued as a suitable psychometric instrument in the psychoeducational field, helping professionals in a school environment to be more aware of the areas of cognitive development in which a student diagnosed with ADHD will have more difficulties, and in doing so, providing more adjusted and effective psychopedagogical measures when it comes to supporting students in their adaptation to the school environment.

### Further Research Directions

For future research, it would be interesting to apply the ENFEN battery at the same time as another instrument of similar characteristics that also evaluate the development of EFs, in order to carry out comparisons between the scores of both tools and degree of correlation in the detection of deficiencies associated with the diagnosis of ADHD. If positive relationships are found between different tests and the ENFEN battery when measuring EFs, this would support and strengthen the data obtained in this study.

## Data Availability Statement

The raw data supporting the conclusions of this article will be made available by the authors, without undue reservation.

## Ethics Statement

The studies involving human participants were reviewed and approved by Ethics Committee of the University of Alicante, assigning it file number UA-2018-03-08. Written informed consent to participate in this study was provided by the participants’ legal guardian/next of kin.

## Author Contributions

IN-S provided the project idea, article concept, and design; planned the timeline; substantially involved in the data, material, and article acquisition; mainly responsible for drafting, writing, and revising the review article; and responsible for selection and final approval of the scholarly publication. RL-C provided substantial help with the concept and design; contributed substantially to the project by drafting and revising the review article; and was responsible for the final approval of the scholarly publication. JG-F provided substantial help with the concept and design; contributed substantially to the project by drafting and revising the review article; and was responsible for the final approval of the scholarly publication. MR-F, CG-G, and RJ-R participated in drafting the work and revising it critically with respect to important intellectual content in all its phases. All authors contributed to the article and approved the submitted version.

### Conflict of Interest

The authors declare that the research was conducted in the absence of any commercial or financial relationships that could be construed as a potential conflict of interest.

## References

[ref1] Abad-Mas L. A. Català O. C. Mulas F. Andrés R. (2017). Comparación entre el diagnóstico del trastorno por déficit de atención/hiperactividad con el DSM-5 y la valoración neuropsicológica de las funciones ejecutivas. Rev. Neurol. 64, 95–100. 10.33588/rn.64S01.2017011, PMID: 28256694

[ref2] American Psychiatric Association (2013). Diagnostic and statistical manual of mental disorders. 5th Edn. Washington, DC: American Psychiatric Association.

[ref3] Ardilla A. Ostrosky F. (2008). Desarrollo histórico de las funciones ejecutivas. Rev. Neurol. 8, 1–21.

[ref4] Artigas J. (2011). ¿Sabemosquées un trastorno? Perspectivas del DSM 5. Rev. Neurol. 52, S59–S69. 10.33588/rn.52S01.2010802, PMID: 21365605

[ref5] Ato M. López-García J. J. Benavente A. (2013). Un sistema de clasificación de losdiseños de investigación en psicología. An. Psicol. 29, 1038–1059. 10.6018/analesps.29.3.178511

[ref6] Baldo J. V. Schwartz S. Wilkins D. Dronkers N. F. (2006). Role of frontal versus temporal cortex in verbal fluency as revealed by voxel-based lesion symptom mapping. J. Int. Neuropsychol. Soc. 12, 896–900. 10.1017/S1355617706061078, PMID: 17064451

[ref7] Barkley R. (1997). Behavioral inhibition, sustained attention and executive functions: constructing a unifying theory of ADHD. Psychol. Bull. 121, 65–94. 10.1037/0033-2909.121.1.65, PMID: 9000892

[ref8] Barkley R. A. (2006a). Attention-deficit hyperactivity disorder: A handbook for diagnosis and treatment. New York: Guilford Press.

[ref9] Barkley R. A. (2006b). There relevance of the still lectures to ADHD: a commentary. J. Atten. Disord. 10, 137–140. 10.1177/1087054706288111, PMID: 17085623

[ref10] Barkley R. A. (2009). Avances en el diagnóstico y subclasificación del trastorno por déficit de atención/hiperactividad: qué puede pasar en el futuro respecto al DSM-V. Rev. Neurol. 49, 101–106. 10.33588/rn.48S02.2009003, PMID: 19280563

[ref11] Bausela-Herreras E. Tirapu-Ustárroz J. Cordero-Andrés P. (2019). Executive function deficits and neurodevelopmental disorders in childhood and adolescences. Rev. Neurol. 69, 461–469. 10.33588/rn.6911.2019133 31762001

[ref12] Bradley J. D. D. Golden C. J. (2001). Biological contributions to the presentation and understanding of attention-deficit/hyperactivity disorder: a review. Clin. Psychol. Rev. 21, 907–929. 10.1016/S0272-7358(00)00073-8, PMID: 11497212

[ref200] Brown T. E. (2003). Trastornos por déficit de atención y comorbilidades en niños, adolescentes y adultos. Barcelona: Masson.

[ref13] Bustillo M. Servera M. (2015). Análisis del patrón de rendimiento de una muestra de niños con TDAH en el WISC-IV. Rev. Psicol. Clin. Niños Adolesc. 2, 121–128.

[ref14] Corral S. Arribas D. Santamaría P. Sueiro M. J. Pereña J. (2005). Escala de Inteligencia de Wechsler para niños-IV. Madrid: TEA Ediciones.

[ref15] Coslett H. B. Bowers D. Verfaellie M. Heilman K. M. (1991). Frontal verbal amnesia: phonological amnesia. Arch. Neurol. 48, 949–955. 10.1001/archneur.1991.00530210075027, PMID: 1953420

[ref16] Craig F. Margari F. Legrottaglie A. R. Palumbi R. Giambattista C. Margari L. (2016). A review of executive function deficits in autism spectrum disorder and attention-deficit/hyperactivity disorder. Neuropsychol. Dis. Treat. 12, 1191–1202. 10.2147/NDT.S104620, PMID: 27274255 PMC4869784

[ref17] Devena S. Watkins M. (2012). Diagnostic utility of WISC-IV general abilities and cognitive proficiency index difference scores among children with ADHD. J. Appl. Sch. Psychol. 28, 133–154. 10.1080/15377903.2012.669743

[ref18] DuPaul G. J. Power T. J. Anastopoulos A. D. Reid R. (1998). ADHD rating scale—IV: Checklists, norms, and clinical interpretation. New York: Guilford Press.

[ref19] Evans J. D. (1996). Straightforward statistics for the behavioural sciences. Washington: Thomson Brooks/Cole Publishing Co.

[ref20] Feinberg T. E. Farah M. J. (1997). The development of modern behavioral neurology and neuropsychology. Behavioral neurology and neuropsychology. New York: McGraw-Hill.

[ref21] Fenollar J. Navarro N. González C. García J. (2015). Cognitive profile for children with ADHD by using WISC-IV: subtype differences? Rev. Psicodidáct. 20, 157–176. 10.1387/revpsicodidact.12531

[ref22] Fernández M. L. Castellanos R. B. Pérez J. P. Arias R. M. (2013). Estudio de las funciones ejecutivas en diabetes tipo 1 mediante el test de evaluación neuropsicológica de las funciones ejecutivas en niños (ENFEN). An. Pediatr. 78, 88–93. 10.1016/j.anpedi.2012.05.005, PMID: 22727598

[ref23] Frazer A. Gerhardt G. A. Daws L. C. (1999). New views of biogenic amine transporter function: implications for neuropsychopharmacology. Int. J. Neuropsychopharmacol. 2, 305–320. 10.1017/S1461145799001625 11285147

[ref24] Frazier T. W. Demaree H. A. Youngstrom E. A. (2004). Meta-analysis of intellectual and neuropsychological test performance in attention-deficit/hyperactivity disorder. Neuropsychol. 18:543. 10.1037/0894-4105.18.3.543, PMID: 15291732

[ref25] Fuster J. M. (1997). The prefrontal cortex: Anatomy, physiology and neuropsychology of the frontal lobe. New York: Lippincott-Raven.

[ref26] Gallego V. Hernández-Mendo A. Reigal R. Juárez R. (2015). Efectos de la actividad física sobre el funcionamiento cognitivo en preadolescentes. Apunts. Educ. Física Deportes 121, 20–27. 10.5672/apunts.2014-0983.es.(2015/3).121.03

[ref27] García T. González-Castro P. Pérez C. R. Cueli M. García D. Á. Álvarez L. (2014). Alteraciones del funcionamiento ejecutivo en el trastorno por déficit de atención con hiperactividad y sus subtipos. Psicol. Educ. 20, 23–32. 10.1016/j.pse.2014.05.003

[ref800] Goldberg E. (2004). El cerebro ejecutivo. 2nd Edn. Barcelona: Masson.

[ref28] Golden C. J. (2001). Stroop: Test de colores y palabras. 3rd Edn. Madrid: TEA Ediciones.

[ref29] Goldman M. D. (1984). The frontal lobe: uncharted provinces of the brain. Trends Neurosci. 7, 425–429. 10.1016/S0166-2236(84)80147-2

[ref30] Gupta R. Kar B. R. (2009). Development of attentional processes in ADHD and normal children. Prog. Brain Res. 176, 259–276. 10.1016/S0079-6123(09)17614-8, PMID: 19733762

[ref31] Gupta R. Kar B. R. (2010). Specific cognitive deficits in ADHD: a diagnostic concern in differential diagnosis. J. Child Fam. Stud. 19, 778–786. 10.1007/s10826-010-9369-4

[ref32] Gupta R. Kar B. R. Srinivasan N. (2009). Development of task switching and post-error-slowing in children. Behav. Brain Funct. 5, 1–13. 10.1186/1744-9081-5-38, PMID: 19754947 PMC2751760

[ref33] Gupta R. Kar B. R. Srinivasan N. (2011). Cognitive-motivational deficits in ADHD: development of a classification system. Child Neuropsychol. 17, 67–81. 10.1080/09297049.2010.524152, PMID: 21140311

[ref34] Gupta R. Kar B. R. Thapa K. (2006). “Specific cognitive dysfunction in ADHD: an overview” in Recent developments in psychology. eds. Mukherjee J. Prakash V. (New Delhi: DIPR), 153–170.

[ref35] Heaton R. K. (1981). Wisconsin card sorting test manual. Odessa, FL: Psychological Assessment Resource Inc.

[ref36] Henry J. D. Crawford J. R. (2004). A meta-analytic review of verbal fluency performance in patients with traumatic brain injury. Neuropsychol. 18:621. 10.1037/0894-4105.18.4.621, PMID: 15506829

[ref37] Henry L. A. Messe D. J. Nash G. (2015). Executive functioning and verbal fluency in children with language difficulties. Learn. Instr. 39, 137–147. 10.1016/j.learninstruc.2015.06.001

[ref38] Hojat M. Xu G. (2004). A visitor’s guide to effect sizes–statistical significance versus practical (clinical) importance of research findings. Adv. Health Sci. Educ. 9, 241–249. 10.1023/B:AHSE.0000038173.00909.f6, PMID: 15316274

[ref39] Huang F. Sun L. Qian Y. Liu L. Ma Q. G. Yang L.. (2016). Cognitive function of children and adolescents with attention deficit hyperactivity disorder and learning difficulties: a developmental perspective. Chin. Med. J. 20, 549–558. 10.4103/0366-6999.187861, PMID: 27503016 PMC4989422

[ref40] Juneja M. Mehar H. Sairam S. Verma N. Jain R. Mishra D. (2019). Children’s Color Trail Test for objective assessment of attention in children with attention deficit hyperactivity disorder: a diagnostic accuracy study. Indian Pediatr. 56, 1025–1028. 10.1007/s13312-019-1684-2, PMID: 31884432

[ref41] Kasper L. J. Alderson R. M. Hudec K. L. (2012). Moderators of working memory deficits in children with attention-deficit/hyperactivity disorder (ADHD): a meta-analytic review. Clin. Psychol. Rev. 32, 605–617. 10.1016/j.cpr.2012.07.001, PMID: 22917740

[ref42] Lavigne R. (2009). Comparación de tratamiento farmacológico, intervención psicoeducativa y tratamiento combinado en niños con TDAH: la importancia del Sistema Ejecutivo y sus Funciones. tesis doctoral. Málaga: Facultad de Ciencias de la Educación, Universidad de Málaga.

[ref43] Lavigne R. Romero J. F. (2010a). El TDAH: ¿quées?, ¿qué lo causa?, ¿cómo evaluarlo y tratarlo? Madrid: Pirámide.

[ref44] Lavigne R. Romero J. F. (2010b). Modelo teórico del trastorno por déficit de atención con hiperactividad I: definición operativa. Electron. J. Res. Educ. Psychol. 8, 1303–1338. 10.25115/ejrep.v8i22.1417

[ref45] Lezak M. D. (1982). The problem of assessing executive functions. Int. J. Psychol. 17, 281–297. 10.1080/00207598208247445

[ref46] López-Villalobos J. A. Serrano-Pintado I. Andrés J. L. Sánchez-Mateos J. D. Alberola-López S. Sánchez-Azón M. I. (2010). Utility of the Stroop test in attention deficit/hyperactivity disorder. Rev. Neurol. 50, 333–340. 10.33588/rn.5006.2009418 20309831

[ref47] Lozano N. Ruival P. Riva S. Mancilla M. Alvarez L. Dhers P. (2015). Evaluación de las Funciones Ejecutivas de niños entre 6 y 12 años: normalización de la Batería Neuropsicológica ENFEN en la zona sur de la Provincia de Buenos Aires. Hologramática 22, 49–71.

[ref48] Luria A. R. (1980). Fundamentos de neurolingüística. Barcelona: Toray-Masson.

[ref49] Martín R. González P. A. Izquierdo M. Hernández S. Alonso M. A. Quintero I.. (2008). Evaluación neuropsicológica de la memoria en el trastorno por déficit de atención/hiperactividad: papel de las funciones ejecutivas. Rev. Neurol. 47, 225–230. 10.33588/rn.4705.2008140, PMID: 18780266

[ref50] Mattison R. E. Mayes S. D. (2012). Relationships between learning disability, executive function, and psychopathology in children with ADHD. J. Atten. Disord. 16, 138–146. 10.1177/1087054710380188, PMID: 20837980

[ref51] Mayes S. D. Calhoun S. L. (2006). WISC-IV and WISC-III profiles in children with ADHD. J. Atten. Disord. 9, 486–493. 10.1177/1087054705283616, PMID: 16481665

[ref400] Mummery C. J. Patterson K. Hodges J. R. Wise R. J. (1996). Generating “tiger” as an animal name or a word beginning with T: differences in brain activation. Proc. B Bio. Sci. 263, 989–995. 10.1098/rspb.1996.0146, PMID: 8805836

[ref52] Navarro I. Fenollar J. Carbonell J. Real M. (2020). Memoria de trabajo y velocidad de procesamiento evaluado mediante WISC-IV como claves en la evaluación del TDAH. Rev. Psicol. Clin. Niños Adolesc. 7, 23–29. 10.21134/rpcna.2020.07.1.3

[ref53] Navarro I. Fenollar J. Lavigne R. Juárez R. (2017). Detecting differences between clinical presentations in ADHD through the cognitive profile obtained from WISC-IV. Univers. J. Psychol. 5, 179–186. 10.13189/ujp.2017.050403

[ref54] Navarro I. Real M. Lavigne R. García J. M. Piqueras J. M. (2019). Predictive capacity of the Spanish neuropsychological assessment of executive functions battery when diagnosing child ADHD. Rev. Lat. Am. Psicol. 51, 153–161. 10.14349/rlp.2019.v51.n3.2

[ref55] Pedreira J. Peña M. S. (2011). Evaluation of the control of interference in children with ADHD using the five-digit test. Rev. Invest. Divulgación Psicol. Logop. 2, 31–37.

[ref56] Pelegrín C. Tirapu J. (1995). Neuropsiquiatría del daño prefrontal traumático. Monogr. Psiquiatr. 7, 11–21.

[ref57] Peña-Casanova J. Casals-Coll M. Quintana M. Sánchez-Benavides G. Rognoni T. Calvo L.. (2012). Estudios normativos españoles en población adulta joven (Proyecto NEURONORMA jóvenes): métodos y características de la muestra. Neurología 27, 253–260. 10.1016/j.nrl.2011.12.019, PMID: 22397892

[ref58] Pineda D. A. (2000). La función ejecutiva y sus trastornos. Rev. Neurol. 30, 764–768. 10.33588/rn.3008.99646, PMID: 10893741

[ref59] Portellano J. A. (2014). Neuropsychology of attention, executive functions and memory. Madrid: Síntesis.

[ref60] Portellano J. A. Martínez-Arias R. Zumárraga L. (2009). ENFEN: Evaluación Neuropsicológica de las Funciones Ejecutivasen Niños. Madrid: TEA Ediciones.

[ref61] Price B. H. Daffner K. R. Stowe R. M. Mesulam M. M. (1990). The comportamental learning disabilities of early lobe damage. Brain 113, 1383–1393. 10.1093/brain/113.5.1383, PMID: 2245302

[ref62] Qian Y. Shuai L. Chan R. C. Qian Q. J. Wang Y. (2013). The developmental trajectories of executive function of children and adolescents with attention deficit hyperactivity disorder. Res. Dev. Disabil. 34, 1434–1445. 10.1016/j.ridd.2013.01.033, PMID: 23474996

[ref63] Ramos C. Pérez C. (2017). Inhibitory control and monitoring in children with ADHD. Av. Psicol. Lat. Am. 35, 117–130. 10.12804/revistas.urosario.edu.co/apl/a.4195

[ref300] Reitan R. M. Wolfson D. (1992). Conventional intelligence measurements and neuropsychological concepts of adaptive abilities. J. Clin. Psychol. 48, 521–529. 10.1002/1097-4679(199207)48:4<521::AID-JCLP2270480414>3.0.CO;2-C 1517447

[ref64] Rey A. (2009). REY. Test de copia de una figura compleja. Madrid: TEA Ediciones.

[ref65] Reyes S. Barreyro J. P. Injoque-Ricle I. (2014). Evaluación de componentes implicados en la Función Ejecutiva en niños de 9 años. Cuad. Neuropsicol. 8, 44–59.

[ref66] Rognoni T. Casals-Coll M. Sánchez-Benavides G. Quintana M. Manero R. M. Calvo L.. (2013). Estudios normativos españoles en población adulta joven (proyecto NEURONORMA jóvenes): normas para las pruebas Stroop Color-Word Interference Test y Tower of London-Drexel University. Neurología 28, 73–80. 10.1016/j.nrl.2012.02.009, PMID: 22652138

[ref67] Sánchez M. Lavigne R. Romero J. F. Elósegui E. (2019). Emotion regulation in participants diagnosed with attention deficit hyperactivity disorder, before and after an emotion regulation intervention. Front. Psychol. 10:1092. 10.3389/fpsyg.2019.01092 31178779 PMC6543912

[ref68] Sedó M. A. (2007). The five-digits test. Madrid: TEA Ediciones.

[ref69] Servera M. Cardó E. (2007). ADHD Rating Scale-IV en una muestra escolar española: datos normativos y consistencia interna para maestros, padres y madres. Rev. Neurol. 45, 393–399. 10.33588/rn.4507.2007301, PMID: 17918104

[ref70] Shuai L. Daley D. Wang Y. F. Zhang J. S. Kong Y. T. Tan X.. (2017). Executive function training for children with attention déficit hyperactivity disorder. Chin. Med. J. 130, 549–558. 10.4103/0366-6999.200541, PMID: 28229986 PMC5339928

[ref71] Thaler N. Allen D. McMurray J. Mayfield J. (2010). Sensitivity of the test of memory and learning to attention and memory deficits in children with ADHD. Clin. Neuropsychol. 24, 246–264. 10.1080/13854040903277305, PMID: 19859854

[ref72] Thaler N. Bello D. Etcoff L. (2013). WISC-IV profiles are associated with differences in symptomatology and outcome in children with ADHD. J. Atten. Disord. 17, 291–301. 10.1177/1087054711428806, PMID: 22286109

[ref73] Tirapu J. Cordero P. Luna P. Hernández P. (2017). Propuesta de un modelo de funciones ejecutivas basado en análisis factoriales. Rev. Neurol. 2, 75–84. 10.33588/rn.6402.2016227 28075001

[ref74] Tirapu J. Muñoz J. M. Pelegrín C. (2002). Funciones ejecutivas: necesidad de una integración conceptual. Rev. Neurol. 7, 673–685. 10.33588/rn.3407.2001311 12080519

[ref75] Wechsler D. (2011). WISC-IV escala de inteligencia de Wechsler para niños. Manual técnico y de interpretación. Madrid: Pearson.

[ref76] World Medical Association (2013). World Medical Association Declaration of Helsinki: ethical principles for medical research involving human subjects. J. Am. Med. Assoc. 310, 2191–2194. 10.1001/jama.2013.281053, PMID: 24141714

[ref77] Yasumura A. Omori M. Fukuda A. Takahashi J. Yasumura Y. Nakagawa E.. (2019). Age-related differences in frontal lobe function in children with ADHD. Brain Dev. 41, 577–586. 10.1016/j.braindev.2019.03.006, PMID: 30952459

